# Single trial prediction of self-paced reaching directions from EEG signals

**DOI:** 10.3389/fnins.2014.00222

**Published:** 2014-08-01

**Authors:** Eileen Y. L. Lew, Ricardo Chavarriaga, Stefano Silvoni, José del R. Millán

**Affiliations:** ^1^Defitech Chair in Non-Invasive Brain-Machine Interface, Center for Neuroprosthetics, School of Engineering, Ecole Polytechnique Fédérale de LausanneLausanne, Switzerland; ^2^Laboratory for Experimental Research on Behavior, Institute of Psychology, University of LausanneLausanne, Switzerland; ^3^Laboratory of Robotics and Kinematics, I.R.C.C.S. S. Camillo Hospital FoundationVenice, Italy

**Keywords:** stroke, self-paced voluntary movement, movement-related potentials, EEG, movement direction, brain-machine interface

## Abstract

Early detection of movement intention could possibly minimize the delays in the activation of neuroprosthetic devices. As yet, single trial analysis using non-invasive approaches for understanding such movement preparation remains a challenging task. We studied the feasibility of predicting movement directions in self-paced upper limb center-out reaching tasks, i.e., spontaneous movements executed without an external cue that can better reflect natural motor behavior in humans. We reported results of non-invasive electroencephalography (EEG) recorded from mild stroke patients and able-bodied participants. Previous studies have shown that low frequency EEG oscillations are modulated by the intent to move and therefore, can be decoded prior to the movement execution. Motivated by these results, we investigated whether slow cortical potentials (SCPs) preceding movement onset can be used to classify reaching directions and evaluated the performance using 5-fold cross-validation. For able-bodied subjects, we obtained an average decoding accuracy of 76% (chance level of 25%) at 62.5 ms before onset using the amplitude of on-going SCPs with above chance level performances between 875 to 437.5 ms prior to onset. The decoding accuracy for the stroke patients was on average 47% with their paretic arms. Comparison of the decoding accuracy across different frequency ranges (i.e., SCPs, delta, theta, alpha, and gamma) yielded the best accuracy using SCPs filtered between 0.1 to 1 Hz. Across all the subjects, including stroke subjects, the best selected features were obtained mostly from the fronto-parietal regions, hence consistent with previous neurophysiological studies on arm reaching tasks. In summary, we concluded that SCPs allow the possibility of single trial decoding of reaching directions at least 312.5 ms before onset of reach.

## 1. Introduction

Brain machine interfaces (BMI) have been recently used for direct control of neuroprostheses by patients with different levels of motor disabilities (Hochberg et al., 2012; Collinger et al., 2013; Courtine et al., 2013; Leeb et al., 2013). In addition, BMI could also be used to improve the efficiency of post-stroke functional training through the use of brain signals to complement impaired muscle control in movement-assisted rehabilitation therapy (Daly and Wolpaw, 2008; Ang et al., 2011; Niazi et al., 2012; Biasiucci et al., 2013; Ramos-Murguialday et al., 2013). Earlier detection of movement intention could possibly minimize the delays in device activation, which may result in a more natural coupling between the motor planning activity in the cortex and the movement-assisted devices (Krebs et al., 2003; Muralidharan et al., 2011). This form of therapy has the potential of speeding up recovery by enhancing the regeneration and reorganization of brain neuronal structures (i.e., brain plasticity) after stroke (Schaechter, 2004; Dobkin, 2007; Kwakkel et al., 2008). For this reason, we are motivated to study how early before the actual movement, the intention to reach toward a target (in the form of discrete direction planning) can be decoded from brain activity. The primary focus of this paper is on single trial decoding of self-paced reaching movements by stroke patients and able-bodied subjects.

Different studies in human and non-human primates have shown the possibility to decode movement parameters from single unit neural activity—such as hand position, velocity, gripping force and muscular activity—for the control of computer cursors and robot arms (Wessberg et al., 2000; Serruya et al., 2002; Taylor et al., 2002; Carmena et al., 2003; Schwartz, 2007; Ganguly and Carmena, 2009; O'Doherty et al., 2011; Hochberg et al., 2012; Collinger et al., 2013). A number of recent studies have proposed the use of non-invasive methods, in particular the electroencephalography (EEG) signal, for decoding reaching directions (Mehring et al., 2003; Waldert et al., 2008; Ince et al., 2010) and continuous trajectories (Wolpaw and McFarland, 2004; Bradberry et al., 2010). Nevertheless most of these studies, in particular those focused on decoding movement direction (Connolly et al., 2003; Mehring et al., 2003; Musallam et al., 2004; Rickert et al., 2005; Rizzuto et al., 2005; Hammon et al., 2008; Waldert et al., 2008; Robinson et al., 2013), rely on cue-based protocols (i.e., where a “go” cue is used to instruct the subject to perform the movement at a fixed time). In contrast, we focus on self-paced reaching, where movements are initiated by the subject in a spontaneous manner without any external cue. This form of reaching movement can better reflect natural motor behavior in humans. Throughout this paper, we define the state prior to movement onset as the *intention* to reach. Intention can be defined as an early plan to move (Andersen and Buneo, 2002) and represents a high level state which specifies the goals of movements rather than the exact muscle activations required for execution. Decoding of intention offers the capability to predict the timing (Niazi et al., 2011; Lew et al., 2012a,b; Xu et al., 2014) and, as studied in this work, the desired target.

Reaching is a complex spatial problem where different reference systems are involved in coding the hand positions directed toward the target location (Philipona et al., 2003; Beurze et al., 2006). Information about the upper limb position, eye position, and target location are combined, coordinated and integrated into a common distributed spatial representations in order to perform a successful goal-directed reach. The posterior parietal cortex (PPC) plays an important role in such coordinate transformation between different reference frames for planning a movement (Cohen and Andersen, 2002). The role of integration is played by a network involving the frontal and parietal cortices for the control and execution of reaching movements, as shown by studies with non-human primate performing visually guided movements (Burnod et al., 1999; Battaglia-Mayer et al., 2003; Gottlieb, 2007). More recently, studies with human subjects using fMRI have shown a similar frontal-parietal network (Culham and Valyear, 2006; Filimon, 2010). These studies suggest that brain signals in the frontal and parietal regions carry the necessary information for decoding visually guided reaching movements (Blohm et al., 2009; Andersen et al., 2010).

We have previously followed a data-driven approach to investigate the contribution of EEG slow cortical potentials (SCPs) in decoding self-paced movement intention (intent to move vs. intent not to move) of both able-bodied subjects and stroke patients (Lew et al., 2012a). Similar conclusions were also obtained by using intracortical recordings (Lew et al., 2012b). Interestingly, it has also been shown that the amplitude of motor cortical local field potentials (LFPs) in lower frequencies (<13 Hz) is modulated with the direction of movement (Rickert et al., 2005). In this work, we evaluate whether the same approach based on SCP allows decoding movement directions prior to actual execution of reaching. We also compare the decoding performance of the EEG activity in different frequency bands. To the best of our knowledge, there is no previous attempt to decode directions of self-paced movements from non-invasive signals before actual movement onset.

## 2. Materials and methods

We analyzed scalp EEG data recorded from three stroke patients and two able-bodied subjects. Participants were instructed to perform a center-out upper limb reaching task. All procedures were approved by the Ethics Committee of the San Camillo Hospital before the experiment. Subjects were informed about the procedures and gave their consent.

Table 1 summarizes the subjects' particulars, including the Fugl-Meyer Motor Assessment score for upper extremity (FMA-UE)—maximum score of 66—for stroke subjects. Patient P1 suffered from a left cerebellar hemorrhagic stroke, also commonly known as intracerebral bleed, where the ipsilateral body part is affected. The second patient P2 suffered from a left nucleo-capsular stroke caused by lesion in a deeper brain structure, thus affecting the contralateral limb. The third patient P3 has had an ischemic stroke caused by lesion in his frontal and left parietal area, thus affecting his right limb. In general, all patients had preserved tactile and proprioceptive sensibility of the arm with normal cognitive abilities at the time of admission to the hospital. All stroke subjects were able to achieve the reaching task without much difficulty, but with significantly longer average reaching time in comparison with the able-bodied subjects (c.f., **Table 6**).

**Table 1:** **Particulars of volunteer experimental subjects**.

**Subject**	**Age**	**Medical condition**	**Dominant hand**	**Paretic arm**	**Time since Stroke**	**FMA-UE**
C1	25	Healthy	Right	–	–	–
C2	26	Healthy	Right	–	–	–
P1	50	Stroke	Right	Left	55 days	56/66
P2	61	Stroke	Right	Right	658 days	43/66
P3	66	Stroke	na	Right	308 days	53/66

### 2.1. Experimental protocols

**Figure 1:**
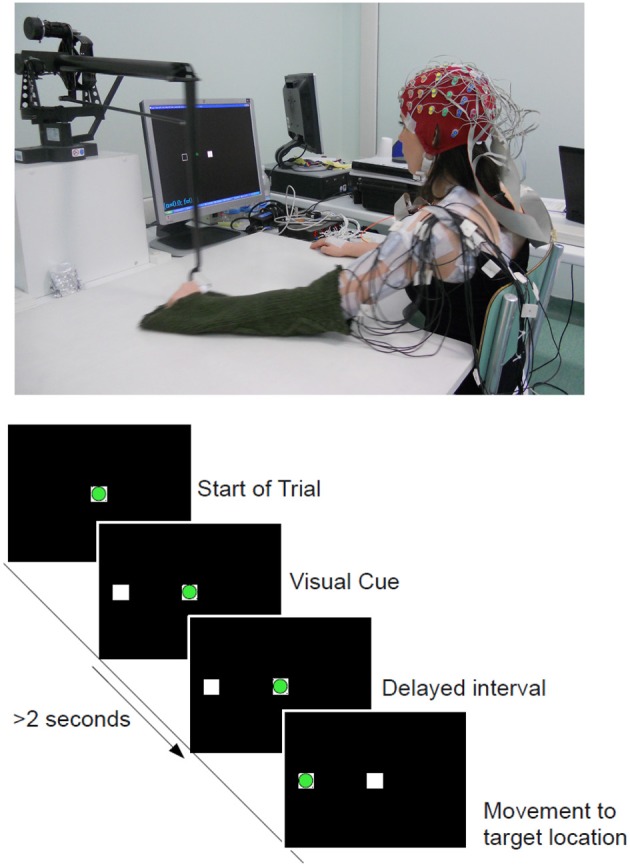
**(Top)** Experimental setup and one of the able-bodied volunteer. **(Bottom)** Experimental Protocol: Timeline of a complete trial. The circle in green refers to the cursor being controlled with the manipulandum and the white squares corresponds to the home **(Center)** and target **(Left)** locations. Movement onset is defined as the time when the green cursor exits the center square.

Subjects were seated in front of a computer screen holding on to a haptic manipulandum (PHANTOM Premium 3.0/6DOF, Sensable Technologies) with their arm resting comfortably on the table as shown in Figure 1. The reaching task was performed with both arms and subjects were instructed to move the manipulandum that controls the position of a cursor (a green circle) on a computer screen [c.f. Figure 1(Bottom)]. The resting position is the condition when the green circle remains inside the white box located in the middle of the screen. The task was to bring the cursor to one of the 4 center-out target locations (up, down, left, right, projected as white-frame boxes). The distance from the home position to each target positions was approximately 15 cm. When the target location was cued, the subject was asked to wait at least 2 s before initiating the movement at their own pace in order to induce a self-paced movement. The role of the visual cue was to ensure equal distribution of target locations during the recordings. Accordingly, when subjects moved before 2 s (an immediate reaction, as in cued-based reaction tasks), the trial was stopped and discarded.

Subjects were asked to stay in a relaxed position during this idle period before initiating a reaching whenever they wish. For each subject, there were 3 recordings of 80 trials each (targets locations were randomly selected), thus resulting in a total of 240 trials for each arm movement. After removing early starts and artifacts, an average of 230 trials remained for both hands and subject groups. This is the same experimental protocol where we have demonstrated the detection of movement onset (Lew et al., 2012a). The design of this experiment allows voluntary initiation of movements, in contrast with most cue-based reaction time task protocols where there is a go cue that instructs the subject when to start the movement. It has been reported that there are neurophysiological differences between internally driven and externally cued movement (Thut et al., 2000). A similar protocol has been used to investigate self-paced arm movements with electrocorticography (ECoG) signals (Ball et al., 2009).

### 2.2. Methods

We simultaneously recorded the EEG and electrooculography (EOG) signals with a portable BioSemi ActiveTwo system using 64 electrodes arranged using an extended 10/20 montage at a sampling rate of 2048 Hz, then downsampled to 256 Hz. EOG channels were placed above nasion and below the outer canthi of both eyes in order to capture horizontal and vertical EOG components. To reduce noise contamination, particularly from eye movement artifacts, we performed our analysis using a selection of 34 channels that excluded the peripheral channels and those that exhibited high correlation with the EOG activity (Lew et al., 2012a). The signals recorded from these 34 electrodes were spatially filtered using the common average referencing (CAR) procedure to remove the global background activity (Offner, 1950; Osselton, 1965; Bertrand et al., 1985).

The EEG signals were pre-processed by applying a zero-phase low-pass Butterworth filter (non-causal filter) with cutoff frequency at 120 Hz. The signals were further downsampled to 128 Hz. In order to evaluate direction-related information in different frequency bands, we applied narrow band filters between [0.1–1] Hz for extracting SCP, [1–4] Hz for delta band, [4–8] Hz for theta band, [7–13] Hz for the alpha band, as well as the ranges [13–20] Hz, [20–30] Hz and [30–45] Hz covering beta and gamma activity. For signals below 7 Hz, we directly used time domain features (EEG amplitude). In particular, for the SCP, Garipelli et al. (2013) have compared various spatial and spectral filtering methods to enhance the signal to noise ratio (SNR) of the slow potentials. Their results have shown higher separability index with the use of narrow pass-band filters between [0.1–1] Hz. They have also reported that CAR filter seems to be a better choice than Laplacian filters. For frequency bands above 7 Hz, we extracted the envelope of the filtered signal by taking the absolute value of the real part of the analytic signal, computed using the Hilbert transform. The Hilbert transform is commonly used in calculating instantaneous amplitude and phase at each time point of a narrow band signal and non-stationary time series such as the scalp EEG signal (Huang et al., 1998; Marple, 1999).

To study the temporal characteristics of brain activity preceding movement onset, referred as *intention period*, we analyzed sliding windows of 250 ms overlapping every 62.5 ms in the period from 2 s before the movement onset to 1 s after. In this paper, the time reported always corresponds to the endpoint of these sliding windows. For each of these windows, we applied the Canonical Variant Analysis (CVA), which is a form of feature selection technique that identifies the most relevant features discriminating among classes, thus significantly reducing the dimensionality of the input vector for the classifier. This technique has previously been proven advantageous for BCI (Galán et al., 2007). CVA extracts subject-specific discriminant spatial patterns that maximizes the difference in variance between the 4 center-out directions classes. As it remains unclear the exact time when the intention to reach is made in a self-paced movement, this method can yield information about movement-related modulations in different brain regions during planning and how they evolve over time. We used the features selected from the training dataset (obtained from 5-fold cross validation) to build a classifier (see below). The feature vector consisted of temporal amplitudes from the 10 channels with the highest discriminant power (DP) for each sliding window. We further reduced the data dimensionality by subsampling to 16 Hz for classification, thus forming a vector of 40 features (10 channels × 4 points) within each 250 ms window.

For classification of movement directions, we relied on Linear Discriminant Analysis (LDA). We built a LDA classifier for each time window. LDA is a simple approach to classification where the samples from each class are modeled with a normal distribution and it is assumed that they have the same covariance matrix (Duda et al., 2001). The probability that the correct class is *y* given a sample *x* can be defined using Bayes' rule:

(1) $$P(C=y|x)=\frac{P(x|C=y)P(C=y)}{P(x)}$$

The classification of a sample *x* is given by *argmax*_*y*_*P*(*C* = *y*|*x*) over all classes. Data distribution, for all classes *P*(*x*|*C* = *y*), is assumed to be normal for each class, and is modeled using the same covariance matrix, Σ.

(2) $$P(x|C=y)=\frac{1}{{\left(2\pi \right)}^{\frac{p}{2}}|\Sigma {|}^{\frac{1}{2}}}e^{-\frac{1}{2}{\left(x-\mu_{k}\right)}^{T}\Sigma^{-1}(x-\mu_{k})}$$

Finally, the performance of our method is evaluated using a 5-fold cross validation procedure by maintaining the chronological order when partitioning the training and testing data (Lemm et al., 2006; Bourdaud et al., 2008). This method yields a more realistic estimation of accuracy than random splitting of trials from the entire recording session.

The movement direction decoding accuracy (DA) used in this paper is derived from the confusion matrix, which interprets the relationship between the actual class labels (i.e., 4-class target directions) and the classified label (predicted output), where the sum of the diagonal elements *n*_*ii*_ refers to the correctly classified trials (the actual target location). DA is defined as the ratio between the correct predictions divided by the total number of trials and measures the sensitivity rate. A value of 1 denotes perfect separation between the movement directions.

As mentioned above, we want to evaluate how early before movement onset the movement direction can be predicted. We calculated the chance level by training several classifiers on a randomized permutation of the labels of the training set (10 × 5 folds cross validation). The chance level is derived from the average performance of these classifiers. The chance level is always shown as a red horizontal dotted line in the Results Section.

In addition to assess the sensitivity rate of our classifiers during the intention period, [-2, 1] s around movement onset, we also evaluated its specificity during the idle period where subjects are supposed not to prepare for the reaching movement, namely from 1 s before the visual target cue to 2 s after the cue.

## 3. Results

### 3.1. Predicting movement directions: able-bodied subjects

**Figure 2:**
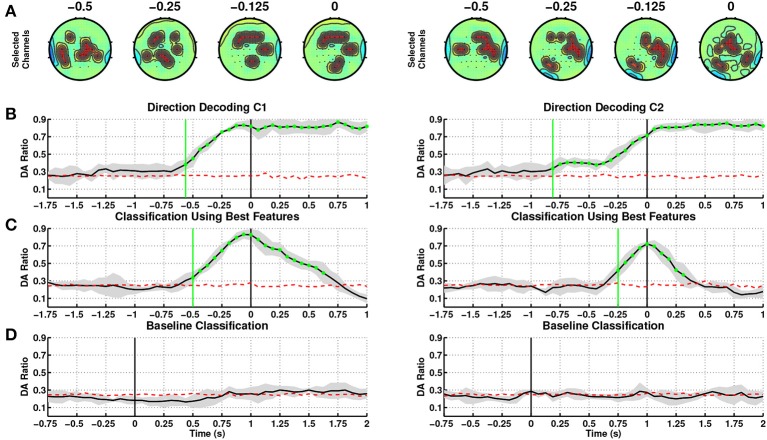
**Decoding of movement direction based on the EEG slow cortical potentials (0.1–1 Hz)**. Able-bodied subjects, C1 (left) and C2 (right) performed the reaching with their dominant arm (right for both). **(A)** Discriminant channels (marked in red) at different time windows of 250 ms (ending at 500, 250, 125, and 0 ms before movement onset), which form a fronto-parietal network. **(B)** Direction decoding performances (DA) using time-specific classifiers during the intention period. *t* = 0 corresponds to movement onset. **(C)** Sensitivity: DA during the intention period obtained using the time-specific classifier with the highest DA. **(D)** Specificity: DA during the idle period. *t* = 0 corresponds to the presentation of the visual cue (c.f. Figure 1).

Figure 2 shows a summary of results obtained from single trial classification of movement directions from SCPs ([0.1–1] Hz) for the able-bodied subjects, C1 and C2, when utilizing their dominant arm (right in both cases). The topographic plots in Figure 2A depict the selected channels based on the ranking of the discriminability power at 500, 250, 125, and 0 ms preceding the onset of movement (channels marked in red refers to the 10 highest ranked channels). These topographic maps show the brain regions which carried the most directional information. Figure 2B shows the average and standard deviation (gray shaded area) of single trial DA of movement direction from time −1.750 to 1 s. We tested if the DA measure is significantly above chance level (shown as red dotted line) with 95% confidence interval using the non-parametric Wilcoxon rank-sum test. The green vertical line in this graph shows the first time when the DA value of a group of five consecutive samples are significantly above chance level (*p* < 0.05).

For subject C1, DA rose above chance level at 687.5 ms before onset (*t* = 0 s) using amplitudes of on-going SCPs, where performance consistently increased until onset of movement and remained at high values afterwards indicating that directional discriminant neural signatures are continuously decoded during movement. The most discriminant channels for this subject are located in the frontal, parietal and ipsilateral regions starting from time 375 to 0 ms prior to onset.

At this stage of analysis we have built classifiers tuned to each time window. This allows us to pinpoint the most pertinent features for decoding movement direction over time. This approach is quite challenging due to the fact that the onset of self-paced movements has a higher variability compared to cue-based movements. In order to explore an online implementation of SCP-based approaches, we have identified that the time window at 62.5 ms *before movement onset* yielded the highest DA (0.83 ± 0.05), and contains the most discriminant features for decoding directions, see Table 2. In this paper, windows after onset are not taken into consideration as they represent movement execution rather than movement intention. We have then used the features and classifier associated to the window with the peak DA to test the decoding performance during the intention period. Figure 2C illustrates the corresponding DA, which climbed above chance level as early as 500 ms before onset. DA increases until 62.5 ms before movement onset, and then decreases after movement onset.

**Table 2:** **Summary of decoding performances before movement onset for able-bodied subjects**.

**Subject ID**	**Arm**	**Highest DA before onset**	**Time of highest DA (ms)**	**Early detection (ms) time-specific classifiers**	**Early detection (ms) selected classifier**
C1	Right	0.83 ± 0.05	−62.5	−687.5	−500.0
	Left	0.75 ± 0.08	−62.5	−437.5	−375.0
C2	Right	0.68 ± 0.05	−62.5	−812.5	−312.5
	Left	0.66 ± 0.08	−62.5	−875.0	−312.5

Time corresponds to the endpoint of the sample window with respect to movement onset (t = 0 ms)

In addition to aim for high sensitivity (high DA) during the intention period, it is also desirable to achieve high specificity (low false positive rate) during the idle period. Figure 2D shows that the selected SCP-based classifier performs at random level during the idle period.

Table 2 summarizes the results of the SCP-based direction decoding for the able-bodied subjects. Regardless of which arm was used, the best decoding performance for both subjects (considering only time before onset) occurred at 62.5 ms before movement onset, with a maximal DA of 0.83 for subject C1. The average DA across folds for each subject was slightly lower for the non-dominant arm. Performances with time-specific classifiers exceeded chance level before onset, early detection, between 875 ms to 437.5 ms and reached DA values above 0.8 after movement onset. For both subjects performance was significantly above random level during the intention period and had a rather low variance. Importantly, performances are quite similar when using the selected time-specific classifier with the best DA (see also Figure 2C). In this case, direction was decoded slightly later—between 500 and 312.5 ms before onset. Also, as shown in Figure 2D, for both subjects and arms DA was at random level during the idle period.

**Figure 3:**
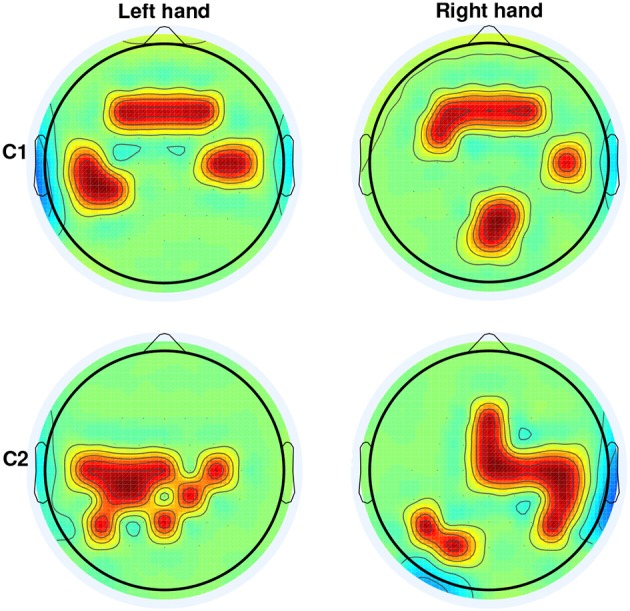
**Selection of channels (in red) from CVA yielding the highest DA for able-bodied subjects C1 and C2 for left and right arm reaching movements**. For both subjects and arms, channels were selected on the window ending at 62.5 ms before movement onset.

Figure 3 depicts the channels selected at the window with the highest DA. They represent the brain regions with highest discriminability power to classify the 4 targets, for the left and right arms of the two able-bodied subjects. Results from both subjects displayed an evident fronto-parietal network, especially when reaching with the right arm. For subject C1, this network is more prone toward the frontal and bilateral central regions when reaching with the left (non-dominant) arm. For subject C2, the ipsilateral central-parietal areas are more discriminant for decoding reaching directions than the contralateral region for the non-dominant. The localization of brain areas will be further analyzed in the Discussion Section.

### 3.2. Predicting movement directions: stroke patients

**Figure 4:**
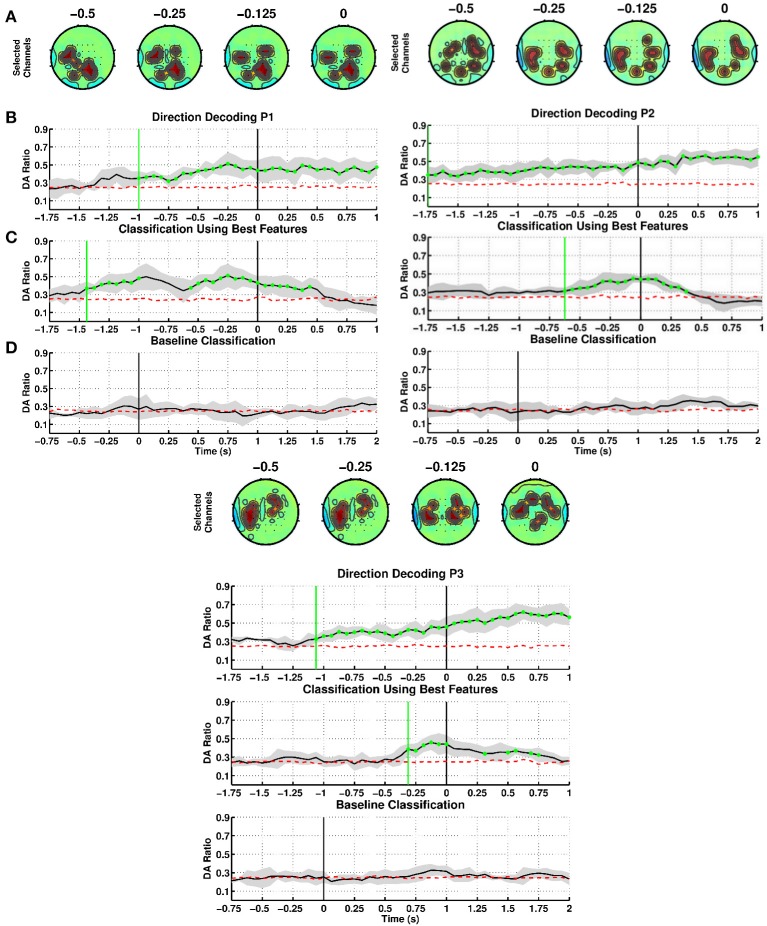
**Decoding of movement direction based on SCPs**. Stroke patients P1, P2, and P3 performed the reaching with their paretic arm. **(A–D)** as in Figure 2.

We evaluated our SCP-based method to decode movement direction when stroke patients performed the reaching task, notably with their paretic arm (see Figure 4). As for able-bodied subjects, we first built time-specific classifiers and then selected the best one (highest DA before movement onset) to test sensitivity and specificity. For patient P1, first panel, the channels selected from SCPs preceding onset were strongly focused at the centro-parietal regions (Figure 4A), with bilateral activation of motor areas toward the time of movement execution. DA of time-specific classifiers (Figure 4B) started to exceed chance level at 1000 ms before onset of movement. The maximum DA was 0.51 at time 250 ms before onset. Using the selected classifier during the intention period (Figure 4C), DA crossed chance level at 1475 ms before onset. However, DA decreased to random level short after and it exceeded chance level again at 550 ms and steadily increased until onset of movement. Thereafter, DA remained above chance till 500 ms after onset. This selected classifier performed at random level during the idle period (Figure 4D).

For patient P2, second panel, CVA selected channels located mainly in the lateral parietal region starting from 500 ms before onset (Figure 4A). Time-specific classifiers reached a peak DA of 0.45 at time 62.5 ms before movement onset, while DA exceeded chance level at 1750 ms before onset (Figure 4B). However, when using the selected classifier, DA crossed chance level at 625 ms before onset (Figure 4C). This selected classifier also performed at random level during the idle period (Figure 4D).

Patient P3, third panel, exhibits similar DA trends to the other patients, although discriminant features were found mostly on the central and frontal areas (Figure 4A). Note that P3 had a frontal and left parietal area lesion. Using time-specific classifiers DA rose above exceeds chance level at 1062.5 ms before onset, peaking at 125 ms before onset with a value of 0.46 (Figure 4B). The selected classifier climbed over chance level at 312.5 ms before movement onset (Figure 4C), while never above random performances during the idle period (Figure 4D).

Table 3 summarizes the results of the SCP-based direction decoding for the stroke patients. The DA values obtained for the paretic arm were above 0.45 for all subjects. This performance was reached between 250 ms to 62.5 ms before onset. Once movement started, DA reached values in between 0.51 and 0.73. Regarding early detection—i.e., when DA exceeded chance level—, time-specific classifiers did it earlier than the selected fixed classifiers (in between −1750 and −1000 ms vs. −625.0 and −312.5 ms, respectively). As for able-bodied subjects, performance was significantly above random level during the intention period and had a rather low variance, for both time-specific and selected classifier. Also, DA was at random level during the idle period. In summary, patients achieved a lower performance than able-bodied subjects, but early detection happened at similar times.

**Table 3:** **Summary of decoding performances before movement onset for stroke patients, paretic arm**.

**Subject ID**	**Paretic arm**	**Highest DA before onset**	**Time of highest DA (ms)**	**Early detection (ms) time-specific classifiers**	**Early detection (ms) selected classifier**
P1	Left	0.51 ± 0.13	−250.0	−1000.0	−1437.5 (−550.0)
P2	Right	0.45 ± 0.04	−62.5	−1750.0	−625.0
P3	Right	0.46 ± 0.08	−125.0	−1062.5	−312.5

Time corresponds to the endpoint of the sample window with respect to movement onset (t = 0 ms)

### 3.3. Direction-related spectral and phasic modulations of EEG activity

**Figure 5:**
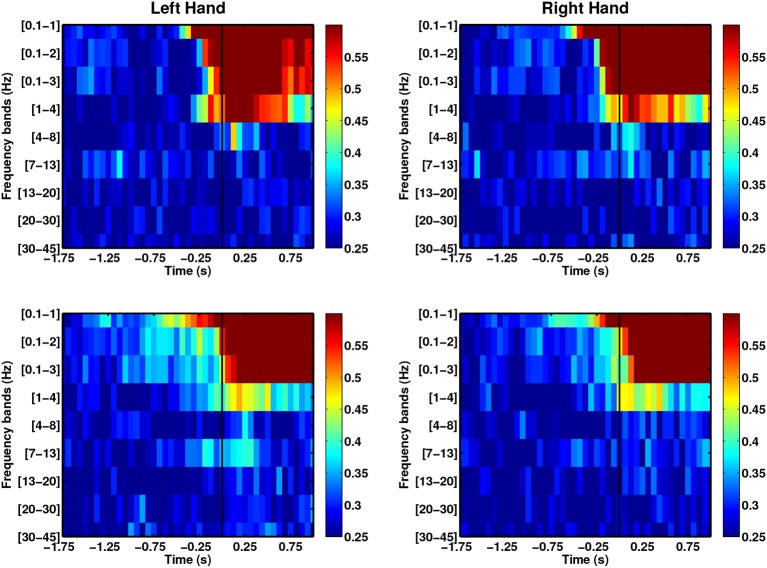
**Comparison of average DAs for different EEG frequency bands for able-bodied subjects who executed left and right hand reach movements**. Both able-bodied subjects (first row: C1, second row: C2) are right-handed. *t* = 0 corresponds to the movement onset.

We evaluated direction-specific modulations in several EEG frequency bands, comprising SCPs (0.1–1 Hz), delta (1–4 Hz), theta (4–8 Hz), alpha (7–13 Hz), beta (13–20 Hz), high beta (20–30 Hz) and low gamma (30–45 Hz). Figure 5 shows the DAs for both left and right arm movements of the able-bodied subjects. The x-axis of each plot corresponds to the endpoint of each decoding window with respect to the movement onset (time = 0 s) and the y-axis provides the frequency bands. We observed direction-specific modulations in both SCPs and delta band activity. In all cases, SCPs showed DAs above chance level before movement onset, although DAs were higher during movement execution. Moreover, narrow band filtered SCPs [0.1–1] Hz seem to provide information that may allow earlier decoding of movement directions as compared to the wider band SCPs. We also observed performances exceeding chance level when signals filtered in the delta band, which has been studied by Waldert et al. (2008) using signals below 4 Hz.

**Figure 6:**
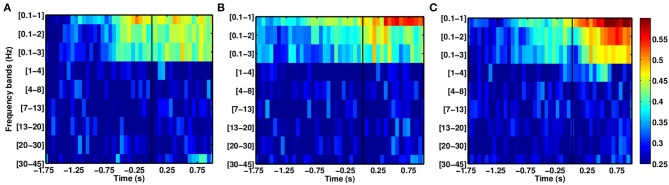
**Comparison of average DAs for different EEG frequency bands for stroke patients who executed reach movements with their paretic arm, (A) P1, (B) P2, and (C) P3**.

In the case of the stroke group (see Figure 6), SCPs yield higher DAs than other frequency ranges. As with the able-bodied subjects, discriminant modulations were observed in SCPs, although the delta band displayed random performance. The DA for the stroke group reached DA values above chance before the able-bodied group during the intention period.

**Figure 7:**
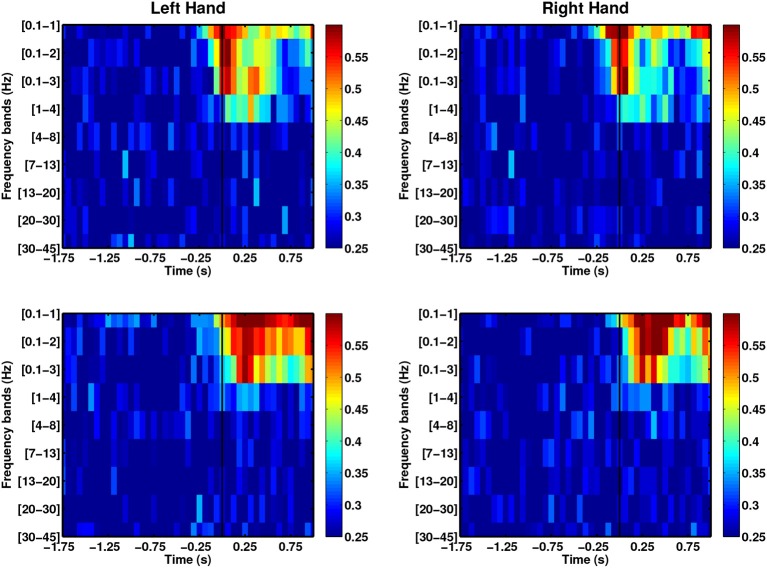
**The use of instantaneous phase features across frequency bands for able-bodied subjects (first row: C1, second row: C2)**.

The use of phase-based features has remained unexplored for decoding movement direction. However, this information has been used in classifying motor imagery-based BCI (Wang et al., 2006; Hamner et al., 2011), auditory target selection (Ng et al., 2013) and decoding continuous movement trajectories (Hammer et al., 2013). We used the instantaneous phase computed using Hilbert transform to explore the decoding power of these features. Figure 7 shows that the DA of phase-based decoding exceeds chance level approximately 250 ms before movement onset for both able-bodied subjects. However, the maximal DA values were lower than when using SCP amplitudes. Analysis from stroke patients showed random decoding performances. Despite this less promising results, the use of phase information can be further explored by studying amplitudes and frequency coupling, as well as entrainment. Indeed, high gamma amplitudes coupled with the phase of low-frequency alpha and theta during waiting (pre-movement) periods in a cued grasping task have been previously used to predict movement types (Yanagisawa et al., 2012). Similarly, Miller et al. (2012) found increased beta-phase entrainment from ECoG signals recorded during the no movement periods in finger flexion.

## 4. Discussion

This preliminary study demonstrates the feasibility of decoding directions of self-pace arm reaching before movement execution from EEG slow cortical potentials. This is also the first time that such a possibility is shown in stroke patients.

Results show good sensitivity (decoding accuracy, DA, significantly above chance level during intention period) and good specificity (DA at chance level during idle period), which are key requirements for its potential use in real-time rehabilitation interventions. This level of specificity indicates that decoding is due to direction-related features from SCPs appearing before onset and not generated by the visual cue. Although promising, the results achieved with stroke patients must be replicated with a larger population. Based on the comparison of different EEG frequency bands (i.e., SCP, delta, theta, alpha, beta, and gamma), we have observed that movement directions can be decoded significantly above chance level using signals filtered at low frequencies (<4 Hz), with SCP yielding the best performance in terms of accuracy and early detection. We have used a systematic approach (previously tested for detecting onset of self-paced movements from invasive and non-invasive recordings) to select discriminant features for decoding reaching movements to four directions at different times before movement onset. The outcome of this feature selection process is used to identify the relevant neural signatures associated to the intention to reach targets at different directions. Consistently with existing literature, we observed a frontal-parietal pattern of activity in the able-bodied group, and a more parietal pattern for the stroke group.

### 4.1. On the role of fronto-parietal networks in the preparation of reaching movements

For both able-bodied subjects, the time-resolved channel selection (Figure 2A) presented a change from an initial bilateral pattern to a dominant ipsilateral activation between 500 and 250 ms prior to movement onset, coinciding with the increase in decoding performance. Activation of the ipsilateral primary motor area seems to be required for the execution of challenging unimanual motor tasks in normal subjects (Roland et al., 1980; Kim et al., 1993; Kobayashi et al., 2003). The selected classifiers contain such ipsilateral primary motor features (Figure 3).

More prominently, our results showed that in the period preceding the movement onset, there is a discriminative pattern involving frontal and/or parietal areas for both able-bodied subjects and stroke patients. Our findings are in agreement with a number of studies, with both humans and non-human primates, where the fronto-parietal brain region seems to play a critical role in planning a reach movement. In a center-out task, Musallam et al. (2004) and Quian Quiroga et al. (2006) studied neural signals related to the goals (direction) of movement from electrodes implanted in the parietal reach region (PRR) of monkeys. Using the memory period activity in a cued paradigm (reflecting monkeys' intent before the “go” signal) from eight PRR neurons, four targets were correctly decoded with 64.4% accuracy.

With respect to human studies, ventral areas of the pre-frontal cortex seem to encode spatial information. Intracranial EEG recordings during a memory task allowed decoding left vs. right target in single-trial movements using either temporal evoked activity or spectral activity with performance between 70 and 80% (Rizzuto et al., 2005). On the other hand, neuroimaging studies showed activation in the PRR, potentially encoding information related to the subject's intention to make a movement toward a particular spatial location (Connolly et al., 2003). Furthermore, using fMRI, Naranjo et al. (2007) showed an evolution of the cortex activation during movement preparation starting from frontal and parietal areas, slowly becoming more focused on the frontal cortex 500ms before movement. Gallivan et al. (2011) decoded grasping top or bottom direction (2-class task) from BOLD signal with accuracy of 55%, which suggested brain activation of the parietal and frontal regions during planning. Most of these studies employed a cue-based paradigm. A similar self-paced study with human ECoG signals has shown a steep rise in decoding accuracy starting from 200 ms before movement onset, peaking at 500 ms post movement (67%), based on spectral amplitude modulations in low frequencies and high gamma band from M1 and pre-motor cortex (Ball et al., 2009). An EEG study on visuomotor adaptation during self-initiated center-out hand movements have shown the involvement the fronto-parietal regions in healthy subjects (Contreras-Vidal and Kerick, 2004). In apparent contrast with our observations, based on the findings from Nenadic et al. (2007) with human intracranial EEG from supplementary motor and parietal areas, the signal in the period 500 ms after the appearance of the target stimulus can be decoded with accuracies of 20% higher than the period before onset in a cue-based protocol.

### 4.2. Performance comparison with previous EEG studies in decoding movement direction

To the best of our knowledge, all previous works aiming at decoding movement directions from non-invasive brain signals (EEG mainly) utilized cue-based protocols. Also, these studies were performed with able-bodied subjects (c.f., Table 4). Column *Type* refers to when decoding was attempted, either before onset (*Intention*) and/or during movement (*Execution*).

**Table 4:** **Non-invasive methods used for decoding movement directions**.

**References**	**Type**	**Directions**	**Features**	**Frequency band**	**Areas**	**Performance**
Waldert et al., 2008	Execution	4	PSD, time-domain	<3 Hz MRP	Motor	MEG: 67.0%, EEG: 55.0%
Hammon et al., 2008	Both	3–4	Time, PSD, wavelet, ICA	High Gamma	Frontal	EEG: 57.0–59.0%
Wang and Makeig, 2009	Intention	2	ICA	<30 Hz	PPC	EEG: 80.25%
Robinson et al., 2013	Both	4	LFC	≤6 Hz	Midline parietal, motor	EEG: 80.0%

In relation to the brain region involved in the execution of reaching movements, Waldert et al. (2008) showed that the motor-related areas are responsible for the execution of reaching from magnetoencephalography (MEG). The other works listed in Table 4 emphasize that decoding of movement direction before onset is correlated to activity in the frontal and parietal areas. Reaching direction planning was decoded using the first 500 ms right after the visual stimulus presentation with performances between 57 and 59% for 4 directions and 80.25% (Hammon et al., 2008) for 2 directions (Wang and Makeig, 2009). Recently, Robinson et al. (2013) reported maximum decoding accuracy of 80% for 4 directions using features extracted from low frequency components of EEG taken from the entire [-1 1]s windows with respect to onset (i.e., including the signal during movement execution). As in our case, they also pointed out to the contribution of SCPs (in particular, motor-related potentials or MRPs)—already known to capture preparation-related modulations—for such decoding.

### 4.3. Role of SCPs in understanding spatial intention

Our results showed that narrow-band SCPs contain information that may allow earlier decoding of movement directions as compared to broad-band SCPs. Such a level of decoding requires proper pre-processing techniques (Garipelli et al., 2013) to enhance the SNR of SCPs through the use of spectral and spatial filters. This finding is consistent with previous works on detection of movement intention (Lew et al., 2012a) and movement execution (Niazi et al., 2012; Robinson et al., 2013; Xu et al., 2014), which showed the advantage of low frequency EEG components.

Surface EEG consists of electrical activity generated by different sources in the active intracranial tissue and negative SCPs reflect the unspecific thalamo-cortical activation of a cortical area (Birbaumer, 1999). The first use of SCPs in BCI was through self-regulation of cortical excitability by a completely paralyzed patient (Kuebler et al., 1998). In recent years, a growing number of studies utilized slow potentials, also known as low frequency component (LFC) of measured neuronal population signals, such as for decoding movement trajectories (Bradberry et al., 2010) and for detecting movement intention (Niazi et al., 2011; Xu et al., 2014). LFCs have also been used in invasive studies exploiting LFPs (Rickert et al., 2005) and ECoG for decoding movement parameters (Milekovic et al., 2012; Hammer et al., 2013). As thalamic activation can be regarded as the allocation of attentional or processing resources toward a specific cortical region, this view has instigated the use of SCPs for studying retention of working memory under the viewpoint of attention (Bosch et al., 2001). The authors reported that retention of spatial locations in working memory was associated with a combination of slow waves over frontal and parietal-occipital sites. Within the framework of our experimental protocol, it seems likely that there were a form of memory retention after the initiation of the visual cue, in the form of spatial memory. A debatable question that follows is the role of working memory in our task and how to better elicit internally-driven intention to prevent the confound of decoding memory retention of the spatial location. This can be accomplished by modifying the experimental design to a self-initiated target selection in order to exclude any form of memory retention. On the other hand, we could explore the feasibility to decode spatial memory retention from EEG signals.

A potential limitation of SCPs for real-time implementation is the significant group delays introduced by filtering, which may be a problem for applications requiring prompt response. Xu et al. (2014) has successfully shown their use in a closed-loop online implementation with true positive rate of 79% at a latency of 315 ms. Further studies could be done to assess this speed-accuracy trade-off for real-time implementation.

### 4.4. Comparing detection of movement intention and prediction of movement direction

Table 5 compares the early detection times (when DA exceeds chance level with performance evaluated using the best classifier) of both the intention to initiate the self-paced movement (Lew et al., 2012a) and the predicted direction. Which component should be detected earlier remains an open question. For all subjects but C1, movement intention was detected earlier than direction. For subject C1, however, detection only differs by 25 ms. Subject P1 deserves an additional comment. Although early detection of direction appeared at −1437.5 ms, decoding was not stable and rapidly decreased to random level. Only at −550 ms direction decoding remained above chance level until movement onset. It seems then that discriminant information about movement onset shortly precedes direction-related information.

**Table 5:** **Comparison between detection of movement intention and prediction of movement direction**.

**Subject**	**Hand**	**Time above chance level (ms)**	
		**Movement intention detection**	**Direction prediction**
C1	Right	−475.0	−500.0
C2	Right	−450.0	−312.5
P1	Left	−600.0	−550.0
P2	Right	−725.0	−625.0
P3	Right	−500.0	−312.5

As a future direction, we will explore the the use both decoders, either in parallel or sequentially, to enhance the reliability of upper-limb neuroprostheses. Fusion of both kind of decoders can be also applied during online operation of the neuroprosthesis so as to achieve continuous control. Another extension is to incorporate eye movements tracking into the trajectory model for decoding directional reaches (Corbett et al., 2012).

### 4.5. Stroke patients and able-bodied subjects

Our results show differences in decoding performance between able-bodied subjects and stroke patients. A reason that could explain this difference is the time required to complete the reaching movement (see Table 6). Able-bodied subjects completed the reaching movement in less than 700 ms, while stroke patients took more than 1000 ms when using their affected limb. These differences were statistically significant (*p* < 0.001, two-tailed Student's *t*-test). These behavioral differences are in line with other studies comparing motor deficits after stroke (Cirstea and Levin, 2000). Despite marked differences in execution times, further analysis showed that trajectories were similarly smooth for able-bodied subjects and stroke patients.

**Table 6:** **Time to complete reaching movements and onset time (interval between target cue presentation to start of movement)**.

**Subject**	**Movement time (ms)**	**Onset time (ms)**
C1	578.09 ± 151.96	3180.53 ± 1439.37
C2	660.40 ± 156.99	2880.47 ± 941.35
P1	3182.64 ± 1129.40	5062.50 ± 3427.20
P2	2333.98 ± 368.24	2728.32 ± 679.00
P3	1169.62 ± 403.50	2810.21 ± 1233.11

*Able-bodied subjects: dominant arm; stroke patients: paretic arm*.

Besides the difference in reaching speed, the age difference between the able-bodied and patient groups could also potentially be a reason for lower decoding performance of approximately 30% by the stroke patients. The issue on age-related differences in BCI performance has been investigated in some studies (Vesco et al., 1993; Friedman et al., 1997; Allison et al., 2010), which reported differences in amplitudes, latencies and scalp topography. Therefore, a fair comparison between able-bodied subjects and stroke patients is only possible using age-matched groups in order to avoid potential confounds. In addition, there are plenty of references to EEG abnormalities caused by cerebrovascular disease (CVD) (Niedermeyer, 1982; Pfurtscheller et al., 1984). These differences are caused by the location, size of damage and time elapsed between stroke and EEG recording, thus resulting in distinct motor-related potentials as compared to able-bodied people (Colebatch, 2007).

Robot-assisted therapy for stroke patients with moderate-to-severe upper-limb deficits has shown promising results in terms of improving motor functional recovery compared to traditional therapy (Kwakkel et al., 2008). Ang et al. (2014) have shown that motor gains obtained with BCI-based therapy were comparable to those attained with intensive robotic therapy. The method proposed in this paper can be further verified in an online implementation to control a robotic arm and, later, in combination with rehabilitation robotics (Krebs et al., 2003) for motor recovery of spinal cord injury and stroke patients^1^. For this purpose, it is important to assess the stability of brain patterns across days (i.e., to determine how MRPs change during the process of functional recovery). Furthermore, in a realistic scenario, it is also important to study the effect of feedback generated by the robot-assisted passive movement on the stability of the brain patterns.

## Funding

This work is supported by the European ICT Programme Project FP7-224631, Swiss NCCR “Robotics,” S. Camillo Hospital Foundation, and Italian Ministry of Health.

### Conflict of interest statement

The authors declare that the research was conducted in the absence of any commercial or financial relationships that could be construed as a potential conflict of interest.

## Footnotes

^1^The University of Texas Health Science Center, Houston, “Brain Machine Interface Control of an Robotic Exoskeleton in Training Upper Extremity Functions in Stroke,” *ClinicalTrials.gov*, April 2014, http://clinicaltrials.gov/ct2/show/NCT01948739
